# Surgical treatment of tension pneumomediastinum in patients with covid-19 at the field hospital: a case series

**DOI:** 10.1186/s13019-022-01966-9

**Published:** 2022-08-24

**Authors:** Phan Quang Thuan, Pham Phan Phuong Phuong, Huynh Phuong Nguyet Anh, Le Phi Long, Le Minh Khoi

**Affiliations:** 1 COVID-19 Intensive Care Center, University Medical Center, Ho Chi Minh City, Vietnam; 2 Department of Cardiovascular Surgery, University Medical Center, Ho Chi Minh City, Vietnam; 3 Department of Critical Care Medicine, University of Medicine and Pharmacy at Ho Chi Minh City, Ho Chi Minh City, Vietnam; 4 Department of Thoracic and Vascular Surgery, University Medical Center, Ho Chi Minh City, Vietnam

**Keywords:** Tension pneumomediastinum, ARDS, COVID-19, Mediastinal drainage, Mechanical ventilation

## Abstract

**Background:**

Tension pneumomediastinum is one of the most serious complications in COVID-19 patients with respiratory distress requiring invasive mechanical ventilation. This complication can lead to rapid hemodynamic instability and death if it is not recognized in a timely manner and intervenes promptly.

**Case presentation:**

We reported 7 COVID-19 patients with tension pneumomediastinum at a field hospital. All patients were critically ill with ARDS. These 7 patients, including 3 females and 4 males in this series, were aged between 39 and 70 years. Tension pneumomediastinum occurred on the first day of mechanical ventilation in 3 patients and later in the course of hospital stay, even 10 days after being intubated and ventilated. The tension pneumomediastinum caused hemodynamic instability and worsened respiratory mechanics with imminent cardiopulmonary collapse. In this series, we used two surgical techniques: (i) mediastinal decompression by suprasternal drainage with or without simultaneous pleural drainage in the first two cases and (ii) mediastinal drainage via suprasternal and subxiphoid incisions in 5 patients. The surgical procedures were feasible and reversed the pending cardiopulmonary collapse. Four patients had a favorable postprocedural period and were discharged from the intensive care center. Both patients undergoing suprasternal drainage died of failed/recurrent tension pneumomediastinum and nosocomial infection. Only one in five patients who underwent mediastinal drainage via suprasternal and subxiphoid incisions died of septic shock secondary to ventilator-associated pneumonia.

**Conclusion:**

Tension pneumomediastinum was a life-threatening complication in critically ill COVID-19 patients requiring mechanical ventilation. Surgical mediastinal decompression was the salvage procedure. The surgical technique of mediastinal drainage via suprasternal and subxiphoid incisions proved an advantage in tension relief, hemodynamic improvement and mortality reduction.

## Background

Tension pneumomediastinum (TPM) is a rare but life-threatening condition in which air accumulates under pressure in the mediastinum. Pneumomediastinum can be primary or secondary [1]. To the best of our knowledge, ten cases of TPM have been reported in COVID-19 patients [2–7]. TPM led to death in 33% of patients and caused permanent hypoxic cerebral damage in 66% of cases without effective intervention. This complication also causes profound hemodynamic instability requiring emergency extracorporeal membrane oxygenation (ECMO) [7].

Based on experiments on a feline model, Macklin and Macklin in 1944 suggested the pathogenesis of pneumomediastinum: high airway pressure caused alveoli to rupture, releasing along the peribronchial and perivascular sheaths to the mediastinum [8]. Until late in 2020, Urigo et al. noted only a few cases of pneumomediastinum in the literature [9]. There are no data on TPM incidence in COVID-19 patients, but we might assume that this condition, also caused by alveolar rupture similar to pneumothorax, increases in those who require invasive mechanical ventilation. It has been shown that although the incidence of pneumothorax is as low as 0.3% in hospitalized COVID-19 patients, this increased to 12.8–23.8% in those with severe respiratory distress requiring invasive mechanical ventilation, with a mortality rate up to 100% [10].

TPM in non-COVID-19 patients can be drained under computerized tomography (CT) guidance [8]. This was not feasible at a field hospital when the number of critically ill COVID-19 patients peaked. There are three different techniques in surgical intervention for TPM: decompression via an incision in the suprasternal notch with blunt finger dissection; the modified Chamberlain procedure; and mediastinal drainage via suprasternal and subxiphoid incisions [5, 11]. The modified Chamberlain procedure was carried out successfully in a COVID-19 patient with TPM [5]. Mediastinal drainage under CT guidance was performed in another patient [6]. Mediastinal drainage via suprasternal and subxiphoid incisions was successful in two COVID-19 patients with TPM [3, 4].

This case series is the first report of surgical mediastinal drainage in Vietnamese COVID-19 patients who were mechanically ventilated and experienced TPM. Of interest, all these cases were carried out at a field hospital during the zenith of the COVID-19 pandemic.

## Case presentation

### Case 1

The first case was a 34-year-old male nonsmoker with no chronic comorbidity except overweight (BMI: 26 kg/m^2^). The patient was admitted to our center in August 2021 following respiratory distress due to a SARS-CoV-2 infection. He was intubated one day after admission because of the worsening acute respiratory distress syndrome. The right pleural drain was inserted on the same day due to the right pneumothorax. We placed him in a prone position with a positive end-expiratory pressure (PEEP) of 12 cmH_2_O, a fraction of inspired oxygen (FiO_2_) of 100%, tidal volume (V_t_) of 8 ml/kg, peak inspiratory pressure (P_peak_) of 40 cmH_2_O, plateau pressure (P_plat_) of 32 cmH_2_O, and static lung compliance (C_stat_) of 12 ml/cmH_2_O. Upon recognition of subcutaneous emphysema during routine physical examination, a chest X-ray was ordered that confirmed 1 mcg/kg/min. We decided to perform left pleural drainage with a 28 F tube and mediastinal decompression via a suprasternal notch incision with blunt finger dissection. After the procedure, his cardiopulmonary status did not improve. The patient died 5 h after drainage (Patient 1 in Table 1).

**Table 1:** Summary of 7 COVID-19 patients with tension pneumomediastinum undergoing surgical mediastinal decompression

Patient	Demographics (Sex, Age, BMI)	Length of MV before TPM (days)	Pre-procedural clinical status	Surgery	Post-procedural clinical status	Outcome
1	Male, 39 yrs, 26 kg/m^2^	8	Prone MV; Nor 1 mcg/kg/min; FiO_2_ 100%; PEEP 12 cmH_2_O; P_peak_ 40 cmH_2_O; P_plat_ 30 cmH_2_O; C_stat_ 12 mL/cmH_2_O; P/F 60	A	Prone MV; Nor 1.2 mcg/kg/min; FiO_2_ 100%, PEEP 12 cmH_2_O; P_peak_ 41 cmH_2_O; P_plat_ 31 cmH_2_O; C_stat_ 12 mL/cmH_2_O; P/F 70	Failure to decompress TPM and the patient died in the same day
2	Male, 69 yrs, 25.5 kg/m^2^	10	Prone MV; Nor 1.2 mcg/kg/min; FiO_2_ 100%, PEEP 12 cmH_2_O; P_peak_ 39 cmH_2_O; P_plat_ 34 cmH_2_O; C_stat_ 11 mL/cmH_2_O; P/F 67	B	Prone MV; vasopressor stopped; FiO_2_ 80%; PEEP 12 cmH_2_O; P_peak_ 38 cmH_2_O, P_plat_ 32 cmH_2_O, C_stat_ 14 mL/cmH_2_O; P/F 75	TPM was initially decompressed, leading to hemodynamic amelioration but then reoccurred. The patient died of TPM and septic shock
3	Female, 69 yrs, 27 kg/m^2^	9	Supine MV; Nor 0.9 mcg/kg/min; FiO_2_ 75%; PEEP 10 cmH_2_O; P_peak_ 31 cmH_2_O; P_plat_ 20 cmH_2_O; C_stat_ 21 mL/cmH_2_O; P/F 78	C	Supine MV; vasopressor stopped; FiO_2_ 40%, PEEP 8 cmH_2_O, P_peak_ 29, P_plat_ 24, C_stat_ 25 mL/cmH_2_O; P/F 115	Drain removal on day 5 No complication. No recurrent TPM. Discharged from hospital after 34 days
4	Male, 70 yrs, 26 kg/m^2^	3	Prone MV; Nor 0.75 mcg/kg/min; FiO_2_ 80%; PEEP 10 cmH_2_O, P_peak_ 35 cmH_2_O, P_plat_ 29 cmH_2_O, C_stat_ 15 mL/cmH_2_O; P/F 90	C	Prone MV; vasopressor stopped; FiO_2_ 45%, PEEP 5 cmH_2_O; P_peak_ 33 cmH_2_O; P_plat_ 26 cmH_2_O; C_stat_ 18 mL/cmH_2_O; P/F 130	Drain removal on day 7. No complication. No recurrent TPM. The patient died of septic shock secondary to VAP
5	Female, 60yrs, 27 kg/m^2^	0	Prone MV; HR 158 bpm, no vasopressor; FiO_2_ 100%; PEEP 10 cmH_2_O; P_peak_ 36 cmH_2_O; P_plat_ 30 cmH_2_O, C_stat_ 14 mL/cmH_2_O; P/F 81	C	Supine MV; HR 118 bpm; no vasopressor; FiO_2_ 60%; PEEP 8 cmH_2_O; P_peak_ 30 cmH_2_O; P_plat_ 28 cmH_2_O; C_stat_ 20 mL/cmH_2_O; P/F 120	Drain removal on day 9. No complication. No recurrent TPM. Discharged from hospital after 36 days
6	Female, 39 yrs, 25.5 kg/m^2^	0	Prone MV; HR 168 bpm; no vasopressor; FiO_2_ 100%; PEEP 14 cmH_2_O; P_peak_ 41 cmH_2_O; P_plat_ 28 cmH_2_O; C_stat_ 17 mL/cmH_2_O; P/F 105; urgent ECMO in preparation	C	Supine VM; HR 120 bpm, no vasopressor; FiO_2_ 60%; PEEP 8 cmH_2_O; P_peak_ 33 cmH_2_O; P_plat_ 26 cmH_2_O, C_stat_ 20 mL/cmH_2_O; P/F 140; ECMO cancelled	Drain removal on day 20. No complication. No recurrent TPM. Transferred to a rehabilitation hospital to wean low flow nasal cannula oxygen after 33 days
7	Male, 49 yrs, 21 kg/m^2^	0	Prone MV; HR 147 bpm; no vasopressor; FiO_2_ 100%; PEEP 10 cmH_2_O; P_peak_ 37 cmH_2_O, P_plat_ 26 cmH_2_O, C_stat_ 19 mL/cmH_2_O; P/F 110	C	Supine MV; HR 110 bpm; no vasopressor; FiO_2_ 60%; PEEP 8 cmH_2_O; P_peak_ 29 cmH_2_O; P_plat_ 25 cmH_2_O; C_stat_ 23 mL/cmH_2_O; P/F 158	Drain removal on day 9. No complication. No recurrent TPM. Discharged from hospital after 62 days

A: Mediastinal decompression via a suprasternal notch incision with blunt finger dissection and pleural drainage. B: Mediastinal decompression via a suprasternal notch incision with blunt finger dissection. C: Mediastinal drainage combining sternal notch and subxiphoid incisions and continuous suction. *BMI* body mass index, *bpm* beats per minute, *C*_*stat*_ static compliance, *ECMO* extracorporeal membrane oxygenation, *FiO*_*2*_ fraction of inspired oxygen, *HR* heart rate, *MV* mechanical ventilation, *PEEP* positive end-expiratory pressure, *P*_*peak*_ peak inspiratory pressure, *P*_*plat*_ plateau pressure, *P/F* PaO_2_/FiO_2_ ratio, *TPM* tension pneumomediastinum

We continued to perform the same technique on another patient (Patient 2 in Table 1). Initially, the procedure successfully decompressed the mediastinum, but TPM reoccurred on postoperative day 5. The patient died on day 7 postoperatively due to recurrent TPM and septic shock.

### Case 2

Following the failure of mediastinal decompression with suprasternal drainage in the first two cases, we switched to mediastinal drainage via suprasternal and subxiphoid incisions in the next 5 cases. We present the first of 5 cases (Patient 3 in Table 1).

**Fig. 1:**
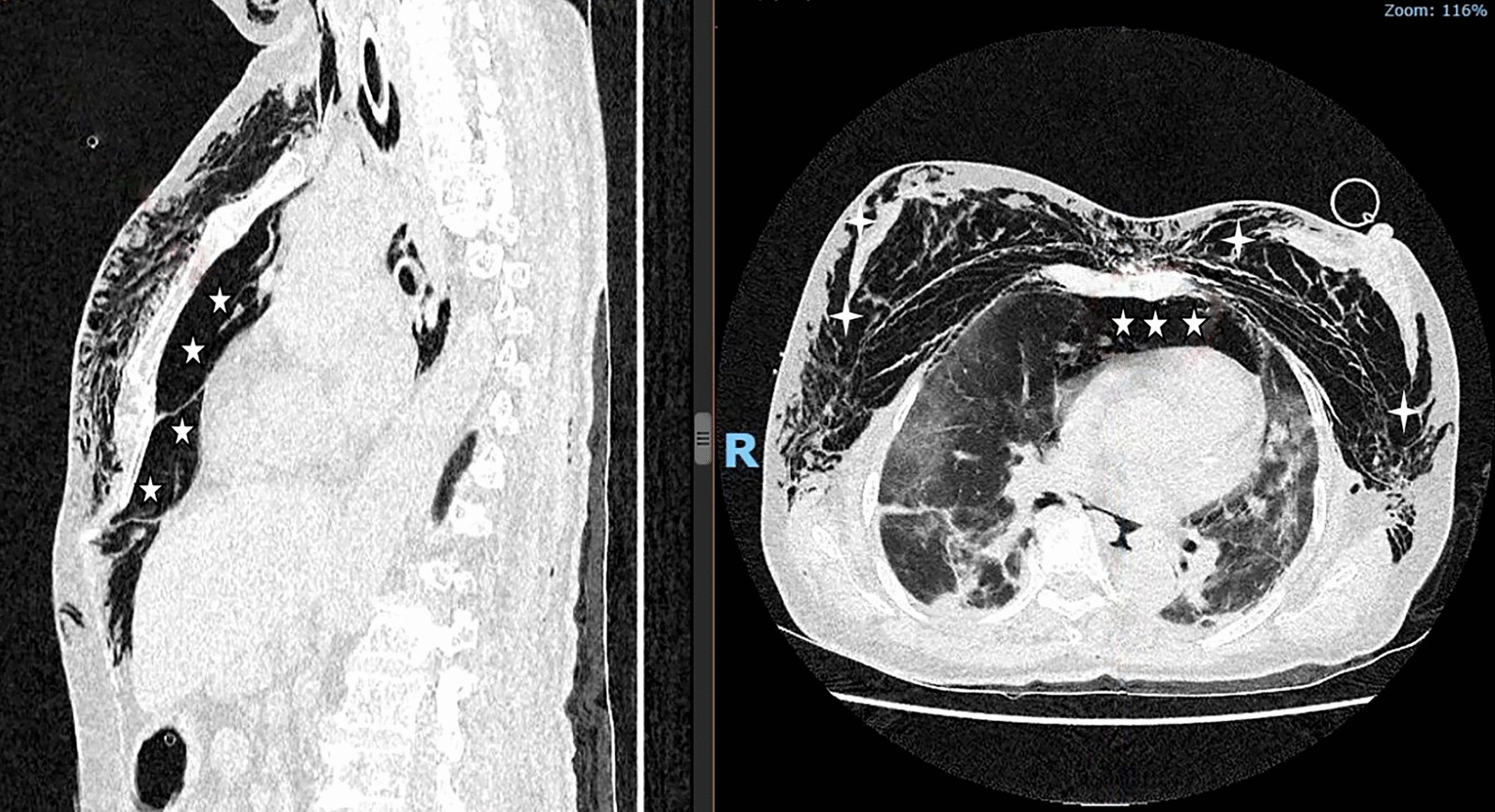
Thorax computed tomography of tension pneumomediastinum before decompression. Left panel: An important accumulation of air in the upper mediastinum extending downward to the preperitoneal cavity (*five-point stars*). Right panel: In addition to the pneumomediastinum, computed tomography showed diffuse subcutaneous emphysema (*four-point stars*)

A 69-year-old female, nonsmoking patient with a BMI of 27 kg/m^2^ and arterial hypertension. She was admitted to our center in September 2021 after a SARS-CoV-2 infection that quickly led to respiratory failure. The patient was put on prone mechanical ventilation for severe ARDS with PEEP 10 cmH_2_O, FiO_2_ 75%, V_t_ 6 ml/kg, P_peak_ 31 cmH_2_O, P_plat_ 20 cmH_2_O, and C_stat_ 21 ml/cmH_2_O. On the 9th day of mechanical ventilation, her hemodynamics became unstable, requiring intravenous noradrenaline at 0.9 mcg/kg/min. Physical examination and chest X-ray confirmed subcutaneous emphysema and pneumomediastinum. The urgent thoracic CT scan revealed a large pneumomediastinum that compressed the heart and superior vena cava (Fig. 1).

**Fig. 2:**
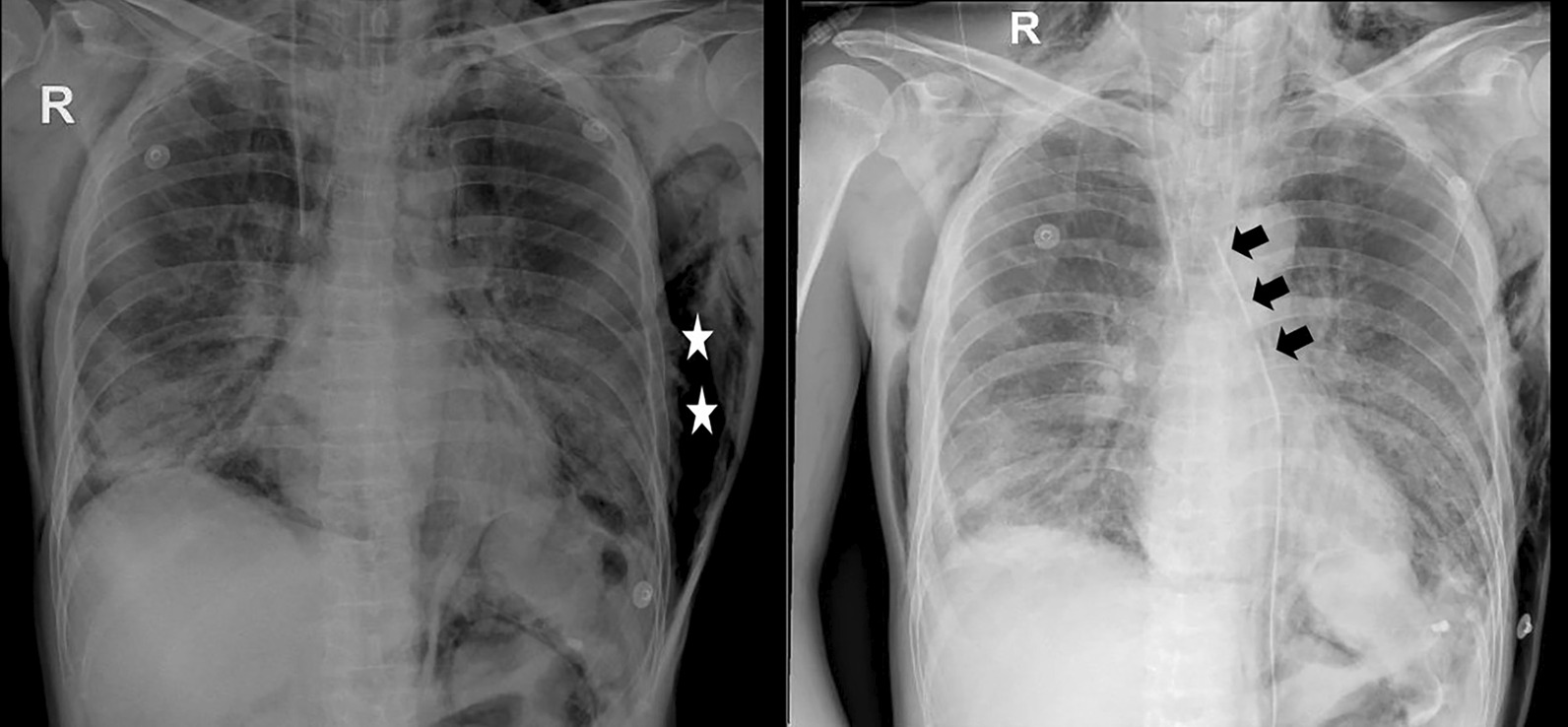
Chest X-ray taken before and after insertion of the drainage tube. Left panel: The chest X-ray taken before drainage shows the presence of remarkable pneumomediastinum. Right panel: The TPM resolved completely after the procedure. The arrowheads indicate the mediastinal drain tube

We performed mediastinal drainage via suprasternal and subxiphoid incisions. A 28 French was inserted and connected with a continuous negative suction at −20 cmH_2_O. Immediately after the procedure, her hemodynamics were stabilized; the ventilator settings improved, with FiO_2_ down to 40%, PEEP 8 cmH_2_O, P_peak_ 29 cmH_2_O, P_plat_ 24 cmH_2_O, and C_stat_ 25 ml/ cmH_2_O. Chest X-ray confirmed a clear reduction in TPM and subcutaneous emphysema (Fig. 2). She was extubated on the day, and the draining tube was removed on day 5 of the procedure. The patient was discharged in good health on day 30 after admission.

This first successful case encouraged us to continue the surgical technique of mediastinal drainage via suprasternal and subxiphoid incisions in the other 4 cases (Patient 4 to Patient 7 in Table 1). Briefly, in all 5 cases, the procedure successfully decompressed the affected mediastinum and prevented recurrent TPM. Four patients experienced a favorable postprocedural stay and were discharged from the COVID-19 intensive care center. Unfortunately, one patient died of septic shock secondary to ventilator-associated pneumonia despite the initial cardiopulmonary improvement.

## Discussion and conclusion

Pneumomediastinum can be primary or secondary. Secondary pneumomediastinum is a well-known complication in ARDS patients undergoing mechanical ventilation [1]. The most dangerous form of pneumomediastinum is life-threatening TPM [9]. The COVID-19 pandemic has increased the number of patients with ARDS requiring mechanical ventilation worldwide and severely affected Vietnam in August and September 2021. As a consequence, the incidence of TPM increases [8]. Reviewing the current medical literature, we found 10 cases of TPM in COVID-19 patients worldwide: 2 cases undergoing mediastinal drainage, 1 case undergoing modified Chamberlain, 1 case undergoing drainage with CT guidance, 3 cases with bilateral pleural drainage, and 3 cases with conservative management. In three patients with conservative management, one died, one had hypoxic encephalopathy, and the last case required ECMO and survived [3–9].

Rapid changes in hemodynamic and respiratory function without apparent cause suggest the occurrence of TPM in a ventilated patient, but imaging confirms the diagnosis. Chest X-ray initially demonstrates the presence of air in the mediastinum and around the heart silhouette. In advanced TPM, a chest X-ray may reveal the earth’s heart sign due to left heart chambers being compressed, leading to reduced filling [9]. Chest CT is the method of choice to visualize the presence of air in the mediastinum and the compressing effects on the heart. The common acoustic windows in echocardiography, when interfered with with air, are also suggestive if clinical manifestations and other complementary examinations are consistent with a pneumomediastinum.

We reported surgical intervention as a rescue treatment for 7 patients with TPM in Vietnam for the first time. The procedure was performed at a field hospital under the restraint of the facility and patient overload. TPM occurred in 1.5% of all patients undergoing invasive mechanical ventilation. There were three female and four male patients in our series. The youngest was 34, and the oldest was 70 years old. A majority of patients (6/7) were obese. The P/F ratio ranged from 60 to 110, indicating moderate to severe ARDS. Prone ventilation was practiced in 6 patients. PEEP at the time of TPM diagnosis ranged from 10 to 14 cmH_2_0. TPM occurred immediately on the first day of mechanical ventilation but was also delayed after 10 days.

**Fig. 3:**
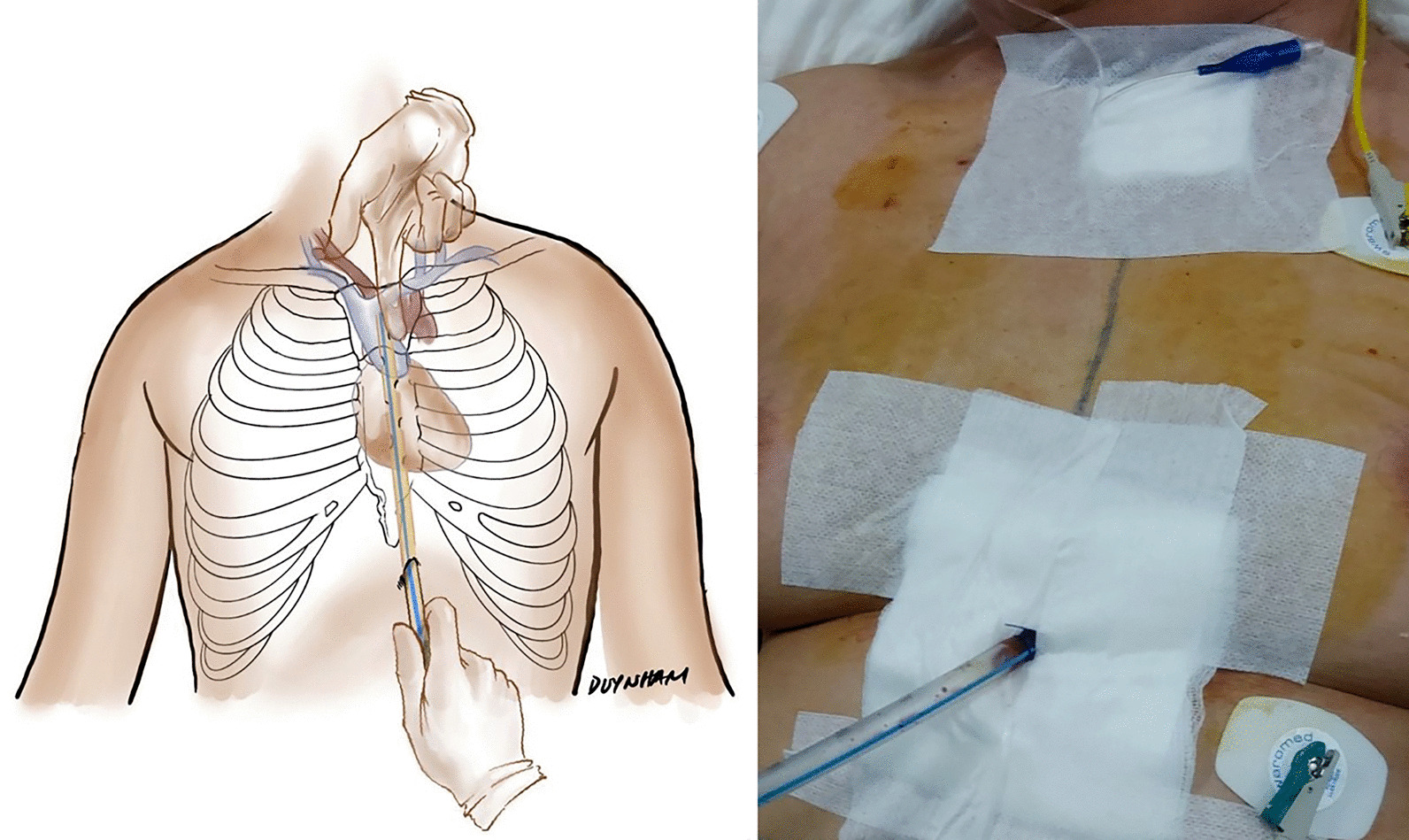
Surgical mediastinal drainage combining suprasternal and subxiphoid incisions. Left panel: The drawing illustrates the technique of inserting the drainage tube. The lower incision is the entrance site of the tube that is advanced upward with the help of the index finger via a suprasternal incision to avoid trauma to the large vessels in the mediastinum. Right panel: The drainage tube is in place

At a field hospital, mediastinal drainage with CT guidance for TPM described by Garcia et al*.* [7] was not feasible, and the recurrence rate was high [11]. The modified Chamberlain procedure [6] was challenging at a field hospital, and the invasiveness is not lower than the technique of sternal notch and subxiphoid incisions. That was why we did not use these techniques in our practice. We performed bilateral pleural drainage and incision in the suprasternal notch with blunt finger dissection in the first patient. The patient died 5 h after the procedure, as the TPM was not resolved. In the second case, we used the same technique of blunt finger dissection without pleural drainage. The procedure was only partially successful in relieving tension, but TPM reoccurred after 5 days. The patient died of septic shock and cardiopulmonary instability secondary to recurrent TPM. After the failure of one suprasternal drainage, we decided to perform mediastinal drainage via two incisions at the suprasternal notch and subxiphoid area and continuous suction (Fig. 3). Technically, the procedure was successful in all 5 patients: the TPM was effectively drained without recurrence. The improvement in haemodynamics in all 5 patients was recognized immediately after the insertion of the draining tubes. An urgent ECMO about to start in one patient was canceled when the collapsed cardiopulmonary system regained normal function quickly after drainage.

Our case series suggests that in COVID-19 patients with TPM, mediastinal drainage via suprasternal and subxiphoid incisions is superior to finger blunt dissection and more feasible than other surgical procedures in the condition of field hospitals. Mediastinal drainage via two incisions at the suprasternal notch and subxiphoid area in COVID-19 patients with life-threatening TPM was feasible and effective in relieving the devastating compression on the cardiopulmonary system. This technique, combined with continuous suction via a draining tube, would prevent recurrent TPM. Medidastinal drainage via suprasternal and subxiphoid incisions can be performed at a field hospital as long as a well-trained and engaged thoracic surgeon is available.

## Data Availability

The clinical dataset used in this study is available from the corresponding author on reasonable request.

## Abbreviations

C_stat_: Static lung compliance
FiO_2_: Fraction of inspired oxygen
PEEP: Positive end-expiratory pressure
P_peak_: Peak inspiratory pressure
P_plat_: Plateau pressure
TPM: Tension pneumomediastinum
V_t_: Tidal volume
